# Comparative Transcriptomic Analysis Reveals Novel Insights into the Adaptive Response of *Skeletonema costatum* to Changing Ambient Phosphorus

**DOI:** 10.3389/fmicb.2016.01476

**Published:** 2016-09-20

**Authors:** Shu-Feng Zhang, Chun-Juan Yuan, Ying Chen, Xiao-Huang Chen, Dong-Xu Li, Jiu-Ling Liu, Lin Lin, Da-Zhi Wang

**Affiliations:** State Key Laboratory of Marine Environmental Science, Department of Environmental Science and Engineering, College of the Environment and Ecology, Xiamen UniversityXiamen, China

**Keywords:** marine diatom, *Skeletonema costatum*, phosphorus, transcriptomics, RNA-Seq, circadian rhythm

## Abstract

Phosphorus (P) is a limiting macronutrient for diatom growth and productivity in the ocean. Much effort has been devoted to the physiological response of marine diatoms to ambient P change, however, the whole-genome molecular mechanisms are poorly understood. Here, we utilized RNA-Seq to compare the global gene expression patterns of a marine diatom *Skeletonema costatum* grown in inorganic P-replete, P-deficient, and inorganic- and organic-P resupplied conditions. In total 34,942 unique genes were assembled and 20.8% of them altered significantly in abundance under different P conditions. Genes encoding key enzymes/proteins involved in P utilization, nucleotide metabolism, photosynthesis, glycolysis, and cell cycle regulation were significantly up-regulated in P-deficient cells. Genes participating in circadian rhythm regulation, such as circadian clock associated 1, were also up-regulated in P-deficient cells. The response of *S. costatum* to ambient P deficiency shows several similarities to the well-described responses of other marine diatom species, but also has its unique features. *S. costatum* has evolved the ability to re-program its circadian clock and intracellular biological processes in response to ambient P deficiency. This study provides new insights into the adaptive mechanisms to ambient P deficiency in marine diatoms.

## Introduction

Phosphorus (P) is an essential macronutrient for phytoplankton growth and proliferation in the ocean (Dyhrman et al., 2007, 2012; White and Dyhrman, 2013; Feng et al., 2015), and its availability is often limiting for primary production (Dyhrman et al., 2007, 2012; Lin et al., 2012; White and Dyhrman, 2013; Feng et al., 2015). Dissolved inorganic phosphorus (DIP) and dissolved organic phosphorus (DOP) are the two major available sources of P in the ocean (Ou et al., 2008; Dyhrman et al., 2012; Lin et al., 2012). DIP is considered to be the only form of P that can be used directly by marine phytoplankton (Ou et al., 2008; Martin et al., 2014). However, its concentration is low, usually less than 0.5 μmol L^-1^, which cannot fulfill the needs of phytoplankton growth (Benitez-Nelson, 2000; Cañellas et al., 2000; Ou, 2006; Ou et al., 2015). DOP, as another important dissolved P pool, comprises a significant portion of total P in both oceanic and coastal waters (Orchard et al., 2009; Ruttenberg and Dyhrman, 2012) and can be utilized by some phytoplankton species (Dyhrman et al., 2012; Feng et al., 2015; Ou et al., 2015). Thus, the capacity of phytoplankton to utilize DOP in a DIP-deficient ambient is essential to their success in the ocean.

Diatoms, as the most abundant and diverse type of phytoplankton, are the major primary producers and an essential component of the food chain (web) in the ocean (Bowler et al., 2010; Obata et al., 2013; Boller et al., 2015; Kim et al., 2015; Zhang H. et al., 2015). As the key player in the marine ecosystem, diatoms play important roles in regulating biogeochemical cycles of biogenic elements, i.e., carbon, silica, nitrogen, and P (Obata et al., 2013; White and Dyhrman, 2013). Moreover, many diatom species are also major causative agents of algal blooms in the ocean (Shatwell et al., 2014; Ou et al., 2015; Zhang Y.J. et al., 2015). Studies show that availability of P influences cell growth, physiological function, and the metabolic activity of diatoms (Dyhrman et al., 2012; Shatwell et al., 2014; Ou et al., 2015). Variations of ambient P also alter the gene expressions patterns of diatoms (Dyhrman et al., 2012; Obata et al., 2013). Multiple response strategies to ambient P deficiency, such as the reallocation of cellular P and the utilization of DOP, are found in P-deficient diatoms (Dyhrman et al., 2006, 2007, 2012; Diaz et al., 2008; Lin et al., 2012; Feng et al., 2015). Recently, the molecular mechanism in response to ambient P change in diatoms has raised concern, and genes or proteins involved in the response to P stress have been identified (Dyhrman et al., 2012; Lin et al., 2012; Alexander et al., 2015; Feng et al., 2015; Ou et al., 2015). However, these studies are focused on limited model diatom species and little effort has been devoted to non-model diatom species with ecological significance. Our molecular-level understanding of diatom response to ambient P change is still very inadequate. Therefore, an in-depth investigation of global gene expression responses of different diatom species to ambient P change may contribute to our understanding of the adaptive mechanisms of diatoms to ambient P variation and stress.

*Skeletonema costatum* is a widely distributed diatom species in the ocean (Boller et al., 2015; Kim et al., 2015; Ou et al., 2015; Zhang H. et al., 2015). Moreover, *S. costatum* often forms intensive blooms, which not only influence biogeochemical cycling but also the food chain (Boller et al., 2015; Zhang H. et al., 2015). Much effort has been devoted to the physiological response of *S. costatum* to ambient P stress. *S. costatum* is able to utilize a wide range of P substrates for growth, including DIP and DOP, and can accumulate polyphosphate in its cells (Miyata et al., 1986; Diaz et al., 2008). It can also respond rapidly to ambient P change (Ou, 2006; Ou et al., 2015), suggesting that it may possess specific adaptive response mechanisms to ambient P deficiency. The molecular mechanisms underpinning these physiological responses are poorly understood. Here, we used RNA-Seq to characterize expression patterns in the transcriptomes of *S. costatum* grown under inorganic P-replete, P-deficient, and inorganic- and organic P-resupplied conditions. The goal of this study was to gain insights into the global regulation of various biological processes in response to P deficiency and resupply.

## Materials and Methods

### Organism and Culture Conditions

The *S. costatum* strain was kindly provided by the Culture Collection Center of Marine Algae, Xiamen University, China. *S. costatum* cells were maintained in K-medium with 48 μM silicon at 20°C under a 14 h:10 h light:dark photoperiod at a light intensity of approximately 100 μmol m^-2^ s^-1^ provided by fluorescent lamps (Keller et al., 1987). Before the experiment, a mixture of antibiotics containing penicillin G (1 g L^-1^), neomycin (250 mg L^-1^), gentamicin (1 g L^-1^), and kanamycin (0.5 g L^-1^) was added to the culture media to eliminate bacterial contamination, and the culture was checked periodically for contamination with 4′,6-diamidino-2-phenylindole stain by microscopic inspection (Cottrell and Suttle, 1993; Ou et al., 2008).

### Experimental Design

The experiment included four treatments: inorganic P-replete, P-deficient, DIP-resupplied, and DOP-resupplied. Each group had triplicate biological repeats. *S. costatum* cells in the exponential growth phase were collected using centrifugation (2,500 *g* for 15 min at 20°C), then washed twice with sterile seawater, and finally cultured in K-medium without P for 2 days to exhaust intracellular P. Then the cultures were inoculated into 12 bottles each with 5 L of culture medium to yield an initial density of 9.0 × 10^3^ cells mL^-1^. In P-replete cultures, 10 μM Na_2_HPO_4_ was added to the culture media at the beginning of the experiment. For P-deficient cultures, 0.2 μM was added to the culture media to maintain cell activity. At day 4, three P-deficient cultures were resupplied with phosphate to the final concentration of 10 μM as the DIP-resupplied group, and another three P-deficient cultures were resupplied with glucose-6-phosphate (G-6-P) to the final concentration of 10 μM as the DOP-resupplied group. The remaining three bottles were maintained as the P-deficient group.

### Physiological Parameter Analysis

Physiological parameters including cell density, the photochemical efficiency of photosystem II (*Fv/Fm*), DIP, DOP, particulate P (PP), and bulk alkaline phosphatase activity (APA) were monitored daily. In addition, samples were collected 4 h and 28 h after the resupply of DIP and DOP.

Three 1 mL aliquots of each bottle were collected every day and fixed in 2% Lugol’s solution for subsequent cell counting under a light microscope (Zhang et al., 2014). The specific growth rate of *S. costatum* was calculated using the following equation: μ = (ln N_2_ – ln N_1_)/(t_2_ – t_1_), where N_1_ and N_2_ were the cell densities at time t_1_ and t_2_ (Ou et al., 2014). Three 5 mL aliquots of each bottle collected at 11:00 am each day were dark acclimated for 15 min and the *Fv/Fm* was measured using PHYTO-PAM (Pulse Amplitude Modulation, ED, Walz, Effeltrich, Germany; Zhang et al., 2007). The bulk APA of *S. costatum* was measured using the method reported by Ou et al. (2015). Cells were collected on pre-combusted GF/F filters (450°C, 2 h) for PP analysis using the method of Solórzano and Sharp (1980), which employs magnesium sulfate (MgSO_4_) and digested for 2 h. The filtrates were used for DIP and DOP analyses; the concentration of DIP was measured using the molybdenum blue method described by Murhpy and Riley (1962) and the concentration of DOP was analyzed using the methods reported by Jeffries et al. (1979). DOP was calculated from the difference between DIP and total dissolved phosphorus (TDP) which employs acid potassium persulfate (K_2_S_2_O_8_; Ou et al., 2015).

### RNA Isolation and Sequencing

*Skeletonema costatum* cells were collected for transcriptomic analyses during exponential growth phase in the P-replete cultures (day 3). The P-deficient cells were harvested on day 4 as well as 4 h and 28 h after the resupply of DIP and DOP. For each sample, cells were collected onto the polycarbonate membrane filter (pore-size 3.0 μm, Millipore) and resuspended in 1 mL Trizol Reagent (Invitrogen, Carlsbad, CA, USA), immediately frozen in liquid nitrogen and subsequently stored at -80°C for RNA isolation (Zhang et al., 2014).

Total RNA was isolated using TRI-Reagent (MRC, Cincinnati, OH, USA) and dissolved in RNase-free water as previously reported by Zhang et al. (2014). For each sample, equal amounts of total RNA from three replicates were mixed together for transcriptome sequencing (Xia et al., 2011; Yu et al., 2016). RNA-Seq libraries were constructed using the Illumina TruSeq^TM^ RNA Sample Preparation Kit (Illumina, San Diego, CA, USA) following the Illumina TruSeq RNA-Seq library protocol. Poly(A) mRNA was enriched using poly-T oligo-attached magnetic beads. First-strand cDNA was synthesized using random oligonucleotides and SuperScript II (Life Technologies, Carlsbad, CA, USA). Second-strand cDNA synthesis was subsequently performed using DNA polymerase 1 (DNAP I; New England BioLabs) and RNase H (Invitrogen; Zhang et al., 2014). In total, one individual paired-end cDNA library was constructed for each set of samples. The cDNA library quality was checked using an Agilent high sensitivity DNA assay on an Agilent Bioanalyzer 2100 system (Santa Clara, CA, USA). Subsequently, the libraries were sequenced on the Illumina Hiseq^TM^ 2000 platform (Expression Analysis Inc., San Diego, CA, USA).

### *De novo* Assembly and Gene Function Annotation

Before assembling, raw reads with adapter contamination, low quality reads, and reads with unknown nucleotides (>5%) were removed. On average, the Q20 percentage of clean reads from all six samples was ∼98. All the downstream analyses were based on clean reads. Next, all the clean reads were assembled as contigs based on the overlap of short reads using the software Trinity *de novo* assembler (Release-20130225^1^) with the min_kmer_cov_set to 1 and all other parameters set to default (Grabherr et al., 2011; Cheng et al., 2014; Zhang et al., 2014).

The unigenes extracted with in-house Perl scripts were aligned against the NCBI-NR database (Release-20130408), NCBI-NT database (Release-20130408), COG (Release-20090331), Swiss-Prot database (Release-63.0), and KEGG database (Release-63.0) using BLAST (v2.2.26 + x64-linux) with a threshold *e*-value ≤ 1e^-5^ as in Zhang et al. (2014).

### Analysis of Differential Expression Genes

The RNA-Seq clean reads of each sample were mapped back to the transcript reference database assembled with Trinity, and the expression levels of unigenes were calculated using the number of mapped reads as EdgeR inputs^2^, followed by normalization of read count number to fragments per kilobase of transcript per million mapped reads (Mortazavi et al., 2008; Cheng et al., 2014; Zhang et al., 2014). Differentially expressed genes (DEGs) were identified through 13 pair-wise comparisons with different physiological/ecological relevance (Supplementary Table S1). According to the comparison method developed by the Beijing Genomics Institute (BGI, China), the probability of one gene being expressed equally between two samples was judged according to the *p*-value corresponding to the differential gene expression test and false discovery rate (FDR; Audic and Claverie, 1997; Benjamini and Yekutieli, 2001; Li et al., 2014). A FDR of 1% or less and a fold change ≥4 were set as the threshold for significant differential expression. DEGs were identified as enriched in GO terms (*p* ≤ 0.05) and metabolic pathways (*q* ≤ 0.05) by searching against GO and KEGG, respectively (Kanehisa et al., 2006, 2008; Sun et al., 2014).

### Validation of the DEGs Using qRT-PCR

The total RNA of each sample used for quantitative RT-PCR (qRT-PCR) analysis was extracted as described above. The expression levels of alkaline phosphatase (*scoap*), inorganic phosphate transporter (*PiT*), phospholipase D (*PLD*), cryptochrome 1 (*cry 1*), and phytochrome B (*phy B*) were examined using qRT-PCR. First-strand cDNA was synthesized using the FastQuant cDNA RT Kit with gDNase (TIANGEN, Beijing, China). qRT-PCR was performed on an ABI 7500 System (Applied Biosystems) using a SuperReal PreMix Plus (SYBR Green) Kit (TIANGEN, Beijing, China). Thermocycling was conducted as follows: 50°C for 2 min, 95°C for 10 min, 40 temperature cycles at 95°C for 30 s, and 60°C for 60 s. The primers designed for qRT-PCR in this study are listed in Supplementary Table S2. Calmodulin (*calm*), a commonly used housekeeping gene (Shi et al., 2013; Zhang et al., 2014), was chosen as the reference gene to normalize the expression of *scoap*, *PiT*, *PLD*, *cry 1*, and *phy B*. Relative expressions of DEGs were calculated based on the 2^-ΔΔCt^ relative response method (Zhang et al., 2014).

### Statistical Analyses

A Student’s *t*-test was performed to compare the differences between the control (P-replete) and each treatment group for cell density, *Fv/Fm*, DIP, DOP, PP, and bulk APA; a *P*-value < 0.05 was regarded as a significant difference. Prior to analysis, data were tested for the equality of variances. All tests were performed using SPSS 17.0 (SPSS Inc., Chicago, IL, USA).

## Results

### Physiological Responses of *S. costatum* to P Nutrient Variations

**FIGURE 1:**
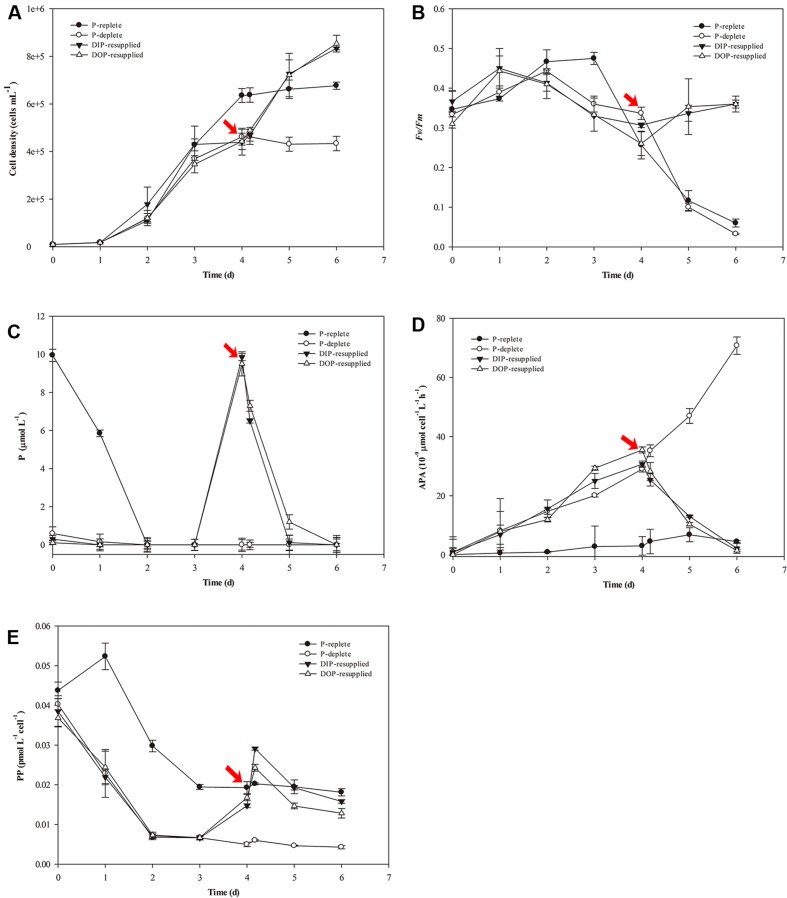
**Physiological parameter variations of *Skeletonema costatum* grown in different P conditions.** **(A)** Cell density, **(B)** *Fv/Fm*, **(C)** P concentration, **(D)** Bulk APA, **(E)** PP content. The red arrow indicates the time point of resupply with Na_2_HPO_4_ or G-6-P. Each data point is the average of three biological replicates, and error bars represent the standard deviation. *Fv/Fm*, maximum chlorophyll fluorescence; Bulk APA, bulk alkaline phosphatase activity; P, including inorganic phosphorus and organic phosphorus in the culture media; PP, particulate organic phosphorus.

Physiological responses of *S. costatum* to P-depletion and -resupply are shown in **Figure 1**. For the P-replete cultures, cell density increased rapidly and reached a peak (6.5 × 10^5^ cells mL^-1^) at day 4, and then entered into stationary phase (**Figure 1A**). In the other three groups with 0.2 μM P, the growth trends were similar to the P-replete group in the first 3 days with low cell density. From day 4, cells in the P-deficient group maintained a stable level with a cell density ∼4.0 × 10^5^ cells mL^-1^. However, growth of P-deficiency cultures recovered rapidly after P resupply, and cell densities reached high levels of 9.0 × 10^5^ cells mL^-1^ at day 6 (**Figure 1A**).

In the P-replete group, *Fv/Fm* reached a peak (∼0.5) at day 3 and then declined gradually to a low value (less than 0.1). *Fv/Fm* of the P-deficient group decreased from day 3 to the end of the experiment. For the P-resupplied group, *Fv/Fm* began to recover after DIP or DOP resupply at day 4. However, *Fv/Fm* of both groups did not reach the maximum value (∼0.5) as that obtained in the P-replete group (**Figure 1B**).

The concentration of soluble reactive P decreased rapidly in all cultures. In the P-replete group, P concentration decreased to 6.0 μM at day 1, and was undetectable at day 2 (**Figure 1C**). However, bulk APA varied a little from day 1 to 6 with a slight increase in the last 2 days (**Figure 1D**). In the other three treatment groups, APA increased from day 1 to day 4 before the resupply of DIP or DOP (**Figures 1C,D**). After resupplement of P, both DIP and DOP concentrations decreased rapidly and DIP was exhausted after 28 h of P-resupply. APA decreased rapidly in both DIP and DOP-resupplied groups until the end of the experiment (**Figures 1C,D**).

Variations of PP contents are shown in **Figure 1E**. PP contents decreased rapidly in the first 3 days in all groups, and then maintained stable levels in both the P-replete and P-deficient groups at day 4, but PP contents in P-deficient cells were much less than those in P-replete cells (**Figure 1E**). After resupply of P, PP contents increased rapidly in P-resupplied-4 h cells but decreased in P-resupplied-28 h cells until the end of the experiment (**Figure 1E**).

There were no significant differences between DIP- and DOP-resupplied groups, indicating that *S. costatum* could utilize both inorganic and organic P as the P source for cell growth.

### RNA-Seq and *De novo* Assembly

In this study, six samples were sequenced with an average read length of 90 bp (**Table 1**), and all clean reads have been deposited in the SRA database of GenBank^3^ with the BioProject accession number PRJNA313486. After removing low quality reads and trimming, poly-N and adapter sequences, the Q20 of the clean reads data ranged from 98.05 to 98.42% and the GC content of either left end or right end read was constant at approximately 47% for each sample. Using Trinity software, these clean reads were *de novo* assembled to 34,942 unique unigenes (transcripts): 31,959 unigenes were obtained from P-replete; 34,590 from P-deficient; 31,530 from DIP-resupplied-4 h; 31,562 from DOP-resupplied-4 h; 34,679 from DIP-resupplied-28 h; and 34,253 from DOP-resupplied-28 h (**Table 1**). The average length of unigenes was 1,430 bp with an N50 length of 2,157 bp (Supplementary Figure S1).

**Table 1:** Summary of *Skeletonema costatum* transcriptome under P-replete, P-deficient, and P-resupplied conditions.

Items	P-replete	P-deficient	DIP-resupplied-4 h	DOP-resupplied-4 h	DIP-resupplied-28 h	DOP-resupplied-28 h
Number of raw reads	55,267,270	54,821,068	54,889,210	57,018,990	56,436,568	55,267,270
Number of clean reads	53,219,614	51,734,734	51,781,332	54,213,018	53,574,486	52,359,168
Q20 percentage (%)	98.35	98.30	98.05	98.39	98.42	98.36
GC percentage (%)	47.97	47.20	47.58	47.22	47.68	47.21
Number of contigs	52,863	58,590	53,809	57,291	54,308	56.456
Number of unigenes	31,959	34,590	31,530	34,679	31,562	34,253


### Gene Function Annotation

Overall, 26,374 unigenes were successfully annotated in at least one database (**Table 2**). A total of 26,102 unigenes presented significant similarity to known proteins in the NCBI-NR database. Among them, 44.6% of the annotated unigenes were matched with sequences from the diatom *Thalassiosira pseudonana* CCMP 1335 and 38.6% with sequences from the diatom *T. oceanica* (Supplementary Figure S2).

**Table 2:** Results of unigene annotation against public databases.

Database	Number of unigenes
NR	26,102
NT	7,260
Swiss-Prot	10,782
KEGG	12,090
COG	11,063
GO	8,289
At least in one database	26,374


**FIGURE 2:**
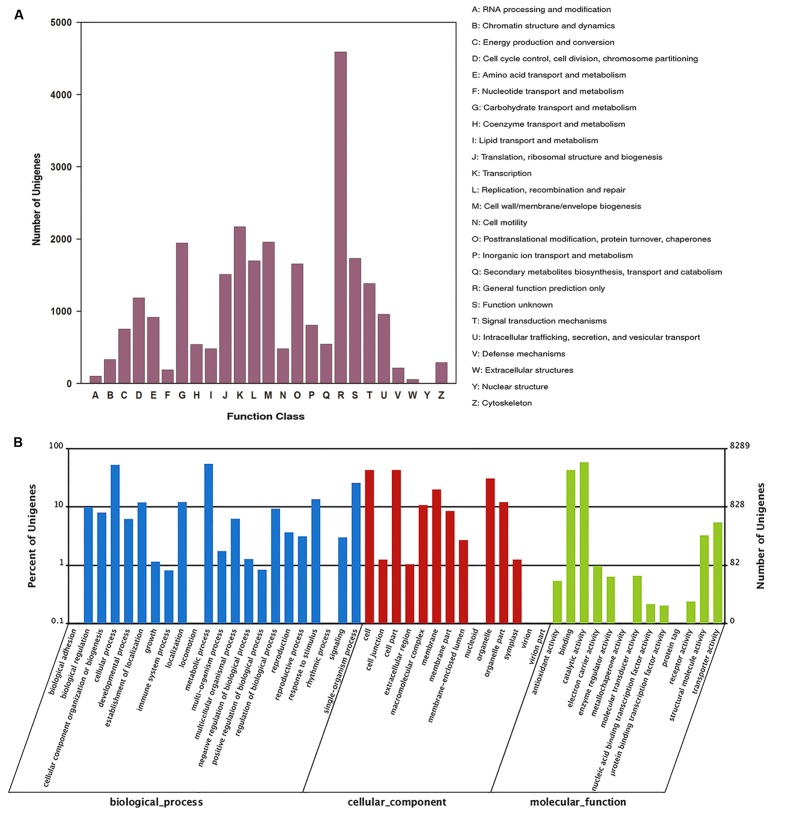
**COG and GO functional classification of all unigenes.** **(A)** COG annotation of unigenes, **(B)** GO categorization of unigenes.

Functional classification of all unigenes was conducted using the COG and GO databases. 11,063 unigenes were annotated against the COG database and classified into 25 categories based on sequence homology (**Figure 2A**). Among these categories, the largest group was the most common and the non-specific category of general function prediction only (41.5%), followed by transcription (19.6%), cell wall/membrane/envelope biogenesis (17.7%), and carbohydrate transport and metabolism (17.6%).

Of the 26,102 unigenes, 8,289 unigenes were classified into three ontologies and 49 sub-categories (**Figure 2B**). Among the ontology of biological processes, metabolic process (54.4%) and cellular process (52.2%) were the two dominant groups, followed by single-organism process (25.7%), response to stimulus (13.6%), localization (12.1%), and establishment of localization (11.9 %).

KEGG annotation revealed that 12,090 unigenes were annotated against the KEGG database (**Table 2**). In the second level, all annotated unigenes were classified into 18 categories (Supplementary Figure S3B), and most of the unigenes were associated with translation (16.5%), carbohydrate metabolism (14.4%), and lipid metabolism (12.6%), followed by nucleotide metabolism (6.3%), environmental adaption (3.1%), and membrane transport (1.0%). The top 25 abundant biochemical pathways with numbers of assigned unigenes are shown in Supplementary Figure S3A.

### Differentially Expressed Genes

**FIGURE 3:**
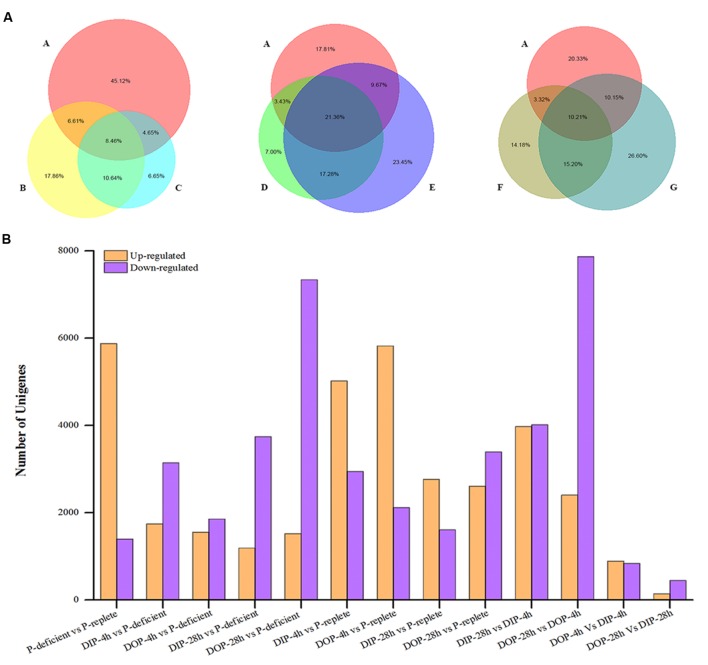
**Statistical analysis of differential expression genes in *S. costatum* under different P conditions.** **(A)** Distribution of differentially expressed genes shared among different *P* conditions. The size of the circle represents the number of differential expression genes. A represents P-replete vs. P-deficient, B represents DIP (dissolved inorganic phosphorus)-supplied-4 h vs. P-deficient, C represents DOP (dissolved organic phosphorus)-supplied-4 h vs. P-deficient, D represents DIP-supplied-28 h vs. P-deficient, E represents DOP-supplied-28 h vs. P-deficient, F represents DIP-supplied-28 h vs. DIP-supplied-4 h, and G represents DOP-supplied-28 h vs. DOP-supplied-4 h. “A vs. B” is A normalized to B. **(B)** Statistical analysis of differential expression gene number in each comparison between two samples. Orange represents the up-regulated genes in the former compared with the latter, and purple represents the down-regulated genes.

Differentially expressed gene were identified through 13 pair-wise comparisons and approximately 20.8% of the unigenes presented significant differential expressions (**Figure 3**). Among them, 16.8% were significantly up-regulated and 4.0% were significantly down-regulated in P-deficient cells compared with P-replete cells. Compared with the P-deficient cells, 14.0% (∼5.0% up-regulated and ∼9.0% down-regulated) and 9.8% (∼4.5% up-regulated and ∼5.3% down-regulated) of the unigenes exhibited significant differences in the DIP-resupplied-4 h and DOP-resupplied-4 h cells while 19.1% of DEGs shared between these two groups and 8.46% of DEGs shared among P-replete, DIP-resupplied-4 h, and DOP-resupplied-4 h (**Figure 3A**). After P-resupply for 28 h, 14.1% of the unigenes (∼3.4% up-regulated and ∼10.7% down-regulated) in the DIP-resupplied-28h cells and 20.4% of the unigenes (4.4% up-regulated and 16.0% down-regulated) in the DOP-resupplied-28 h cells presented significant differences compared with the P-deficient cells, and 12.5% (∼7.9% up-regulated and ∼4.6% down-regulated) and 17.2% (∼7.5% up-regulated and ∼9.7% down-regulated) of unigenes showed significant difference in expression compared with the P-replete cells, indicating that cells had not recovered to normal homeostasis. Compared with the P-deficient cells, 21.36% of DEGs were shared among P-replete, DIP-resupplied-28 h, and DOP-resupplied-28 h, and 38.64% of DEGs were shared between DIP-resupplied-28 h and DOP-resupplied-28 h cells (**Figure 3A**). Comparison of the DIP-resupplied-28 h and DOP-resupplied-28 h cells showed that only a small number of the unigenes presented significant differential expressions (**Figure 3B**).

KEGG pathway analysis revealed that all DEGs were assigned to 124 specific pathways. Among the 15 most frequently represented pathways, the majority of the DEGs were enriched in those pathways which needed P or which were related to cell growth, such as RNA transport, glycerophospholipid metabolism, ether lipid metabolism, nucleotide metabolism, and ribosome biogenesis (Supplementary Table S3; Supplementary Figure S4).

### Transcriptional Regulation of Genes Related to P Utilization

#### Phosphate Transport

Transcripts of three putative phosphate transporters, PiT (major facilitator super-family transporter, phosphate: H^+^ symporter family), solute carrier family 20 (SLC20 family) and solute carrier family 25, member 3 (SLC25A3) were significantly up-regulated in P-deficient *S. costatum* cells by 22-, 5-, and 17-fold, but down-regulated in P-resupplied-4 h cells by 9-, 3-, and 5.0-fold (Supplementary Table S4). After P-resupply for 28 h, transcripts of these phosphate transporters began to increase owing to the exhaustion of ambient P (**Figure 1C**; Supplementary Table S4).

#### P Reallocation

The expression of SPX domain-containing protein involved in vacuolar polyphosphate accumulation increased 23-fold in P-deficient cells and decreased 13-fold in P-resupplied-4 h cells (Supplementary Table S4). However, no significant difference was observed between P-resupplied-4 h cells and P-resupplied-28 h cells.

#### Organic P Utilization

The gene encoding phosphomonoesterase alkaline phosphatase (AP) was up-regulated threefold in P-deficient cells, and down-regulated 39- and 3-fold in DIP- and DOP-resupplied-4 h cells, respectively. Moreover, the transcript of AP was significantly up-regulated 27- and 2-fold when ambient P was almost exhausted after DIP- and DOP-resupply for 28 h, respectively (Supplementary Table S4). In addition, transcripts of several other phosphomonoesterases, such as acid phosphatase, phospholipase A1, and phospholipase B, were all significantly up-regulated in P-deficient cells and down-regulated after P-resupply (Supplementary Table S4). Forty-four unigenes encoding phosphodiesterase of the PLD type were significantly up-regulated in P-deficient cells, and down-regulated significantly in P-resupplied-28 h cells. Transcripts of phosphodiesterase phosphatidylinositol phospholipase C-delta isoform, tyrosyl-DNA phosphodiesterase 1, and glycerophosphoryl diester phosphodiesterase were also up-regulated in P-deficient cells (Supplementary Table S4).

#### Non-P Lipid Utilization

Transcripts of genes involved in sulfolipid biosynthesis, including sulfoquinovosyltransferase, UDP-sulfoquinovose, UDP-sulfoquinovose synthase, and desulfoglucosinolate sulfotransferase, were significantly up-regulated in P-deficient cells and down-regulated after P-resupply (Supplementary Table S4). Moreover, genes encoding betaine lipid synthase involved in betaine biosynthesis presented high expressions in P-deficient cells and low expressions in P-resupplied cells (Supplementary Table S4).

#### Transcriptional Regulation of Genes Related to Circadian Rhythm

**FIGURE 4:**
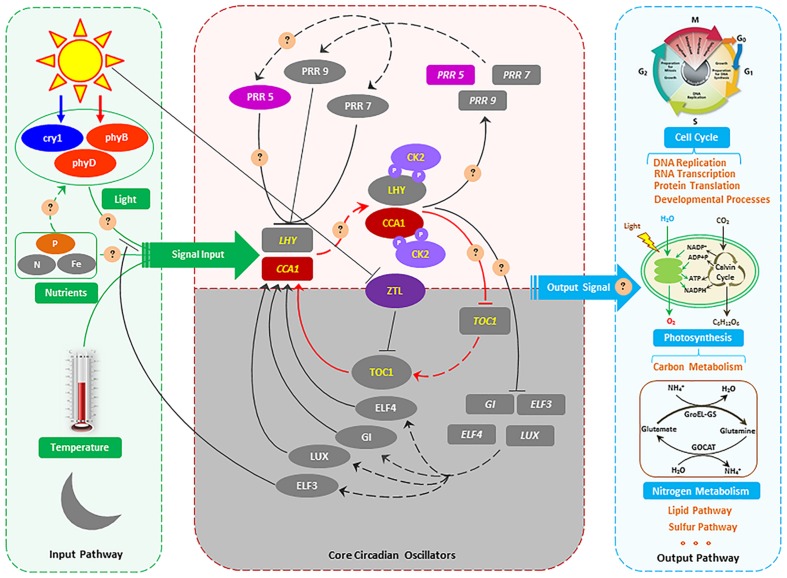
**The proposed molecular model of circadian rhythms in *S. costatum*.** Genes are indicated using oblong shapes with the italic gene names in the shape. Proteins are indicated using oval shapes with the protein name in the shape. Transcription and translation is indicated by dashed lines. Positive action is indicated by solid lines with lines ending in arrowheads, and negative action is indicated with lines ending in perpendicular dashes. The core CCA1 (circadian clock associated 1)/LHY/TOC1 circadian oscillator is highlighted in red lines. Phosphorylation of LHY and CCA1 by CK2 (casein kinase II) is indicated with circled P’s in purple. The shaded area indicates activities peaking in the nighttime, and the white area activities peaking in the daytime. The gray oblong and oval shapes represent the genes or proteins that are identified in *Arabidopsis thaliana* (McClung, 2006; Bujdoso and Davis, 2013), but not identified in the *S. costatum* transcriptome in our study. Because the sampling was conducted in the daytime, so the genes which are active in the nighttime were not detected in this study. In part of the input pathway, light, temperature and nutrient (N, Fe, etc.) have been identified as the main input signal, but P as the input signal has not been reported. For the output pathway, the metabolic pathways affected by circadian rhythms that are demonstrated in the green alga *Chlamydomonas reinhardtii*, the flagellate *Euglena gracilis*, the cyanobacterium *Synechococcus elongata*, and the dinoflagellate *Gonyaulax polyedra* (Hunt and Sassone-Corsi, 2007) and *Arabidopsis thaliana* (Bujdoso and Davis, 2013) might also be affected in *S. costatum*. The circles and questions marks indicate possible regulatory mechanisms of circadian rhythm in *S. costatum* responses to ambient P changing.

Expression of circadian clock associated 1 (CCA1), a gene involved in the circadian control of gene expression, was significantly up-regulated in P-deficient cells and down-regulated after P resupplied (**Figure 4**; Supplementary Table S5). Transcripts of phy B and cry 1 participating in the input pathway of the circadian clock were significantly up-regulated in P-deficient cells and down-regulated in P-resupplied cells (Supplementary Table S5). Moreover, transcripts of casein kinase II (CK2) subunit alpha and beta, ribonuclease P/MRP protein subunit RPP1 (RNase MRP1), and pseudo-response regulator 5 (PRR5) were also up-regulated in P-deficient cells (Supplementary Table S5).

#### Transcriptional Regulation of Genes Related to Other Biological Processes

In the present study, differential expression was detected in genes involved in nucleotide metabolism, glycolysis, photosynthesis, and other important metabolic processes altered with ambient P change. Overall, 77, 46, and 18 DEGs involved in nucleotide metabolism, photosynthesis, and glycolysis were significantly up-regulated in P-deficient cells and down-regulated in P-resupplied cells (Supplementary Table S6). Moreover, transcripts of key proteins regulating the cell cycle, such as cyclin B and cyclin-dependent kinase (CDK), were up-regulated significantly in P-deficient cells and down-regulated in P-resupplied cells (Supplementary Table S6).

#### qRT-PCR Validation of DEGs

**FIGURE 5:**
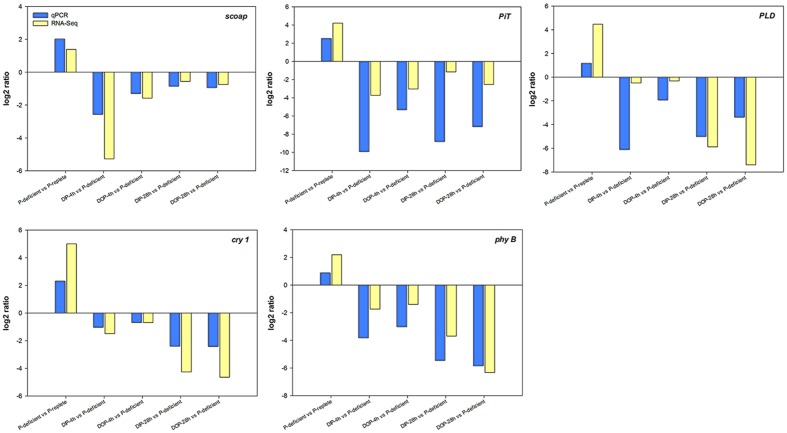
**Quantitative RT-PCR (qRT-PCR) validations of DEGs (differentially expressed genes) in response to P-depletion and resupply.** *calm* was chosen as the internal reference gene.

Five DEGs of *S. costatum* identified in the P-deficient, P-resupplied-4 h, and P-resupplied-28 h groups were selected for qRT-PCR analysis: *scoap*, *PiT*, *PLD*, *cry 1*, and *phy B*. In the P-deficient cells, expression of *scoap* was up-regulated compared with P-replete cells, down-regulated in P-resupplied-4 h cells, and then up-regulated again in P-resupplied-28 h cells (**Figure 5**). Expressions of the other four genes were up-regulated in P-deficient cells and down-regulated in P-resupplied cells (**Figure 5**). It should be pointed out that the correlation between qPCR and RNA-Seq results of all genes were not high owning to the inherent difference between the two methods (**Figure 5**).

## Discussion

Phytoplankton acclimation to ambient P deficiency has been a topic of considerable research and several response strategies have been found in diverse phytoplankton species including diatoms (Dyhrman et al., 2012; Lin et al., 2012; Feng et al., 2015; Ou et al., 2015). We comprehensively compared the transcriptomic responses of *S. costatum* under different P conditions. Though there are no significant differences between DIP-resupplied and DOP-resupplied groups according to physiological responses of *S. costatum* to ambient P variations (**Figure 1**), a considerable number of different DEGs are identified between DOP-resupplied and DIP-resupplied groups (**Figure 3**). These DEGs are significantly enriched in peroxisome, fatty acid metabolism, biosynthesis of secondary metabolites, oxidative phosphorylation, nitrogen metabolism, etc. Here, we focus on key genes involved in adaptation to P deficiency and describe previously unrecognized transcriptional response to ambient P deficiency in *S. costatum*.

### P Utilization in P-deficient Cells

Cells can increase the competitive advantage for phosphate by inducing higher affinity transporters and/or synthesizing more transporters under P-deficient conditions (Dyhrman et al., 2012). In the diatom *T. pseudonana*, a phosphate transporter (PID: 24435) and a high-affinity phosphate transporter are up-regulated at transcription and/or protein level in response to P deficiency (Dyhrman et al., 2012; Fu et al., 2013). The phosphate transporter gene expression pattern of P-deficient *S. costatum* is also consistent with its homolog in *Skeletonema* spp. and *T. rotula* to P-limitation in the field (Alexander et al., 2015). High and low transport systems have also been identified in *S. costatum* (Ou, 2006). In our study, transcripts of phosphate transporters PiT (major facilitator super-family), the SLC20 family and SLC25A3 were significantly up-regulated in P-deficient cells (Supplementary Table S4), and their expression patterns were consistent with the homologs in *Skeletonema* spp. and *T. rotula* in the field (Alexander et al., 2015). As a cell surface transporter, the SLC20 family plays a fundamental housekeeping role in phosphate transport, such as absorbing phosphate from interstitial fluid (Ravera et al., 2007). In plants and fungi, essential transporters for phosphate uptake are proton-coupled transporters of the major facilitator super-family, which also function as transceptors to signal external phosphate concentration (Popova et al., 2010; Nussaume et al., 2011; Pedersen et al., 2013). However, we did not detect any genes encoding high-affinity phosphate transporters in our study. Thus, *S. costatum* might produce different transporters in response to ambient P deficiency, and PiT might play an important role in extracellular P sensing.

In the ocean, polyphosphate is thought to be the product of luxury uptake and storage of phosphate in phytoplankton (Diaz et al., 2008; Dyhrman et al., 2012; Martin et al., 2014). Proteins containing the SPX domain play an important role in maintaining intracellular phosphate homeostasis, and three polyphosphate synthase subunits (vacuolar transporter chaperone 2 (Vtc2), Vtc 3, and Vtc 4) all harbor the SPX domain in yeast (Secco et al., 2012). In our study, the transcript of the SPX domain-containing protein involved in vacuolar polyphosphate accumulation was significantly up-regulated in P-deficient cells (Supplementary Table S4), suggesting that *S. costatum* might have the ability to store P. Our results on intracellular P contents and other physiological responses in P-replete and P-resupplied cells supported this speculation (**Figure 1**). *S. costatum* might increase P allocation to polyphosphate under P deficient conditions, which was consistent with the findings in P-limited *T. pseudonana* (Dyhrman et al., 2012). Our result also supported the view that not all diatom polyphosphate allocation is driven by luxury uptake of phosphate (Dyhrman et al., 2012).

Phytoplankton are able to utilize organic P, and hydrolysis of cell surface P esters by AP is considered to be the most common DOP utilization mechanism (Beszteri et al., 2012; Dyhrman et al., 2012; Lin et al., 2012). In our study, both the activity and the transcript of AP was up-regulated in P-deficient cells (Supplementary Table S4), which was consistent with the findings in *T. pseudonana* and *Karenia brevis* (Dyhrman et al., 2012; Lin et al., 2012). Phosphomonoester can be hydrolyzed directly by a phosphomonoesterase, such as AP, whereas the hydrolysis of high molecular weight phosphodiesters requires phosphodiesterase and polyphosphatase (Cembella et al., 1984; Ou et al., 2015). Genes for several phosphodiesterases were significantly up-regulated in P-deficient cells, especially the PLD (Supplementary Table S4). PLD is a key regulator of cytoskeletal organization and can hydrolyze structural phospholipids (such as membrane lipids), regulating a diverse range of cellular processes, such as membrane transport and cell migration (Pleskot et al., 2013; Frohman, 2015). Its transcription and activity increase upon exposure to various stresses, such as cold, drought, and salinity (Pleskot et al., 2013). Our results indicated that *S. costatum* could utilize both simple organic P (i.e., G-6-P) (**Figure 1**; Supplementary Table S4) and complex organic P (i.e., membrane lipids) as a P source under P-deficient conditions.

Eukaryotic phytoplankton and cyanobacteria are able to replace phospholipid with non-P containing sulfolipids (sulfur containing) and betaine lipids (nitrogen containing) in a P scarce environment to decrease the requirement of cells for P (Yu et al., 2002; Van Mooy et al., 2009; Dyhrman et al., 2012). In our study, transcripts of sulfoquinovosyltransferase, UDP-sulfoquinovose synthase, and desulfoglucosinolate sulfotransferase which are involved in sulfolipid biosynthesis were significantly up-regulated in P-deficient cells (Supplementary Table S4) (Sanda et al., 2001; Yu et al., 2002). Moreover, genes encoding the betaine lipid synthase involved in betaine biosynthesis were also significantly up-regulated in P-deficient cells (Supplementary Table S4). These results indicated that *S. costatum* could utilize non-P containing lipids to reduce the demand for P, which might be an adaptive response of cells to ambient P deficiency. This finding, coupled with a similar response in the diatoms, *T. pseudonana* and *Chaetoceros affinis*, suggests diatoms can utilize non-P containing lipids to reduce their cellular P demand (Van Mooy et al., 2009; Dyhrman et al., 2012).

### Circadian Responses to Ambient P Change

A circadian rhythm occurs ubiquitously in both prokaryotes and eukaryotes driven by a circadian clock, which is entrained by light, temperature, iron, and nitrogen signals in plants (Gutiérrez et al., 2008; Bednářová et al., 2013; Hong et al., 2013; Salomé et al., 2013; Larrondo et al., 2015). Light is regarded as the most important environmental factor involved in resetting the circadian clock (McClung, 2006; Roy and Morse, 2013; Hurley et al., 2014). Studies show that photoreceptors play a significant role in light energy capture and the circadian oscillator mechanism (Bognár et al., 1999). Phytochromes and cryptochromes are the two important photoreceptor families transducting light signal input to the circadian clock (Millar, 2004; McClung, 2006). Furthermore, many light-dependent processes controlled by phytochrome and cryptochrome are also regulated by a circadian rhythm (Millar et al., 1995; Bognár et al., 1999; Millar, 1999). In our study, transcripts of phy B and cry 1 were significantly up-regulated in P-deficient cells and declined rapidly after P resupply (Supplementary Table S5). In *Arabidopsis*, the light period of the phy B-deficient mutant is 1.5–2 h longer than that of the wild type; overexpression (15-fold) of phy B shortens the light period length; cry 1 also plays a similar role as phy B in regulating clock length (Somers et al., 1998). Phy B is the primary high-intensity red light photoreceptor for circadian control, while cry 1 mediates high-intensity blue light signals for the control of period length (Somers et al., 1998). Hence, the light period length might be shortened and the input pathway of the circadian clock influenced in P-deficient *S. costatum* (**Figure 4**).

The core oscillator of the circadian clock is composed of three interlocked feedback loops, and CCA1 is one of the two domain transcription factors (the other is Late Elongated Hypocotyl, LHY) and participates in each loop (**Figure 4**) (McClung, 2006). Furthermore, CCA1 plays a central role in the circadian control of gene expression, and is involved directly in light regulation of the gene expression in plants, providing a molecular link between phytochrome and the circadian oscillator in plant cells (Green and Tobin, 1999). Lack of the CCA1 can cause a shortened circadian period, but its overexpression results in severe disruption of the normal circadian function in plants (Wang and Tobin, 1998; Green and Tobin, 1999; Fujimori et al., 2005; Scaglioni et al., 2008). In our study, the CCA1 gene was up-regulated approximately 1.66-fold in P-deficient cells and down-regulated 1.9-fold after P resupply (Supplementary Table S5). Moreover, genes encoding CK2 subunit alpha and beta and PRR5 were also significantly up-regulated in P-deficient cells, and down-regulated in P-resupplied cells (Supplementary Table S5). CK2 can phosphorylate two components of the central oscillator in *Arabidopsis*, CCA1, and LHY (Sugano et al., 1998; Green and Tobin, 1999; Mulekar et al., 2012). CCA1 phosphorylation by CK2 is necessary for maintaining the normal function of the central oscillator in *Arabidopsis* (Daniel et al., 2004). PRR5 belongs to the PRR family, which is a negative regulator mediating the expressions of CCA1 and LHY genes (Fujimori et al., 2005; Nakamichi et al., 2010; Salomé et al., 2010). It is interesting that overexpression of the PRR5 gene is also related to circadian-associated phenotypes, such as regulation of flowering time (Fujimori et al., 2005). Our results indicated that the phosphorylation of CCA1 was affected by P deficiency, and the core oscillator of the circadian clock was re-programed in response to ambient P change (**Figure 4**).

In our study, transcripts of RNase MRP1 were significantly up-regulated in P-deficient cells, and down-regulated in P-resupplied-28 h cells (Supplementary Table S5). RNase P/MRP, being localized in the nucleolus and cytoplasm, plays an important role in regulating the cell cycle of yeasts (Esakova and Krasilnikov, 2010). RNase MRP mutation occurring in *Saccharomyces cerevisiae* can delay the cell cycle at the end of mitosis (Cai et al., 2002; Esakova and Krasilnikov, 2010). In some unicellular organisms, such as the green alga *Chlamydomonas reinhardtii*, the flagellate *Euglena gracilis*, the cyanobacterium *Synechococcus elongata*, and the dinoflagellate *Gonyaulax polyedra*, cell division is timed by a circadian mechanism (Hunt and Sassone-Corsi, 2007). However, the role of RNase P/MRP in regulating the circadian clock is poorly understood. In general, the circadian rhythm of *S. costatum* was disturbed by ambient P deficiency which might subsequently initiate adaptive mechanisms to the ambient P change in accordance with the signals released by the new circadian clock (**Figure 4**). However, the detailed regulation mechanism still needs further study.

### Other Important Metabolic Processes Responding to Ambient P Change Nucleotide Metabolism

In our study, 33 DNA-directed RNA polymerase (RNAP) I, II, and III genes were up-regulated in P-deficient cells and down-regulated in P-resupplied cells (Supplementary Table S6). DNA-directed RNAPs are complex enzymes containing multiple subunits, i.e., RNAP I, II, III, IV, and V, and are necessary for constructing RNA chains using DNA genes as templates (Jones et al., 1987; Ishihama, 2000; Iyer et al., 2003; Kwapisz et al., 2008). RNAPs regulate the process of gene transcription which allows a cell to acclimate to a changing environment (Ishihama, 2000). However, RNAP genes varied insignificantly in nitrogen-, iron-, or silicon-deficient *T. pseudonana* cells (Mock et al., 2008). These results suggest that variation of RNAP gene expression might be a specific response of diatoms to ambient P-deficiency. In addition, three genes encoding DNAP, including DNAP eta subunit, DNAP alpha subunit A, and DNAP I, were also up-regulated in P-deficient cells and down-regulated in P-resupplied cells (Supplemenatry Table S6). DNAP is an enzyme responsible for DNA replication by assembling nucleotides during cell division. DNAP eta can correct common defects in DNA, particularly important for accurate translesion synthesis of DNA damage resulting from ultraviolet radiation (Goodsell, 2004). Xanthine oxidase and urate oxidase are two key enzymes involved in the degradation of purine bases (Andersen et al., 2006; Abooali et al., 2014). Moreover, transcripts of enzymes involved in the synthesis of nucleotides, such as xanthine oxidase, urate oxidase, GTP synthase, UMP-CMP kinase, phosphoribosylamine-glycine ligase, IMP dehydrogenase, and adenine phosphoribosyltransferase (Van Rompay et al., 1999; Ingerson-Mahar et al., 2010; Sampei et al., 2010), were all up-regulated significantly in P-deficient cells (Supplementary Table S6). These results indicated that P deficiency increased expressions of genes related to DNA damage and RNA biosynthesis, thus the mechanism protecting the nucleotide from damage and maintaining its normal functioning might be initiated.

### Photosynthesis

Phosphorus participates in the co-ordinated regulation of photosynthesis in cyanobacteria (Marcus and Gurevitz, 2000). In our study, expressions of ribulose-bisphosphate carboxylase (RuBisco) large subunit genes increased in P-deficient cells and decreased in P-resupplied-4 h cells (Supplementary Table S6). RuBisco catalyzes the first step of the Calvin cycle of photosynthesis and the oxidation of ribulose bisphosphate in the first step of photorespiration (Miziorko and Lorimer, 1983; Zhang Y.J. et al., 2015). RuBisco usually consists of two types of protein subunit, the large and the small subunits in plants, algae, and cyanobacteria, and the substrate binding sites located in the large subunit. In cyanobacteria, P binds to the RuBisco active site and to another site on the large subunit where it can influence transitions between active and less active conformations of the enzyme (Marcus and Gurevitz, 2000). Transcripts of LHC I, LHC II, P680, and P700 were up-regulated significantly in P-deficient cells, and down-regulated in P-resupplied cells (Supplementary Table S6). The LHC plays an important role in absorbing light and transferring energy to the center of the photosystem (Hiller et al., 1993, 1995; Boldt et al., 2012; Zhang Y.J. et al., 2015). P700 and P680 are the central pigment proteins of photosynthetic system I and II of eukaryotic cells. Furthermore, the expression of genes encoding key photosynthetic proteins, such as photosystem II CP43, photosystem II cytochrome c550 and cytochrome b559, cytochrome b6-f complex iron-sulfur subunit and F-type H^+^-transporting ATPase, were all up-regulated significantly in P-deficient cells and down-regulated in both P-resupplied-4 h and P-resupplied-28 h cells (Supplementary Table S6). These results indicated that P-deficiency enhanced light harvesting and photosynthesis which might be an adaptive mechanism of *S. costatum* to ambient P deficiency. However, diatom photosynthesis is suppressed by ambient nitrate (Bender et al., 2014) or iron limitation (Allen et al., 2008), indicating that photosynthetic responses of diatoms to different nutrients are complicated.

### Glycolysis

In the glycolysis pathway, triosephosphate isomerase is essential for efficient energy production, PGK is a major enzyme in the first ATP-generating step, and phosphofructokinase-1 is also one of the most important regulatory enzymes in glycolysis (van der Kamp, 2013). In our study, genes encoding these enzymes were up-regulated significantly in P-deficient cells. Moreover, genes encoding other important enzymes, such as fructose-1,6-bisphosphatase I, fructose-bisphosphate aldolase, pyruvate kinase, pyruvate dehydrogenase, and aldose 1-epimerase were also up-regulated in P-deficient cells (Supplementary Table S6). However, several genes related to glycolysis are down-regulated in nitrate-limited *T. pseudonana, Fragilariopsis Cylindrus*, and *Pseudo-nitzschia multiseries* (Mock et al., 2008; Bender et al., 2014). These results indicate that responses of diatoms to ambient N or P deficiency are different, which might be caused by their different storage capacity for N and P. In general, glycolysis was significantly enhanced in P-deficient cells in order to produce more energy for cells to acclimate to ambient P deficiency.

### Cell Cycle

Many genes related to cell cycle regulation were identified in the transcriptome of *S. costatum* (Supplementary Table S6). Among them, genes encoding cyclin B and CDK were significantly up-regulated in P-deficient cells and down-regulated in P-resupplied cells (Supplementary Table S6). Cyclin B is a regulatory protein involved in mitosis and contributes to the switch-like all or none behavior of the cell in deciding to commit to mitosis (Zhuang et al., 2013). CDK-cyclin complexes are present in all eukaryotic lineages and play important roles in regulating the cell cycle and ensuring cell division (Robbens et al., 2005; Wang et al., 2013; Zhuang et al., 2013). Overexpression of both genes in P-deficient *S. costatum* suggested that ambient P deficiency disturbed the normal functions of cyclin B and CDK, and subsequently cell division ceased (**Figure 1A**).

## Conclusion

In conclusion, we have shown that *S. costatum* cells initiated multiple adaptive strategies including enhancement of P transport and cellular P reallocation, and utilization of organic P and non-P containing sulfolipids and betaine lipids, in response to ambient P deficiency. Moreover, *S. costatum* cells could re-program the circadian rhythm by up-regulation of the genes involved in the circadian clock, which subsequently triggered the adaptive mechanisms toward ambient P deficiency (**Figure 4**). Overall, this study, to our knowledge, is the first to identify circadian rhythm genes in marine diatoms and to demonstrate that ambient P deficiency could affect cell circadian rhythm. However, whether this type of response to ambient P deficiency is a specific feature of *S. costatum* or is common among a diverse marine algal taxa remains to be elucidated in future.

## Author Contributions

D-ZW, S-FZ, and C-JY planned and designed the research. YC, X-HC, D-XL, J-LL, and LL performed experiments and analyzed data. D-ZW and S-FZ wrote the manuscript.

## Conflict of Interest Statement

The authors declare that the research was conducted in the absence of any commercial or financial relationships that could be construed as a potential conflict of interest.

## Footnotes

1 http://trinityrnaseq.sourceforge.net/

2 http://www.bioconductor.org/packages/release/bioc/html/edgeR.html

3 http://www.ncbi.nlm.nih.gov/sra
