# Supplementary figures and images for: Comparative Transcriptomic Analysis Reveals Novel Insights into the Adaptive Response of Skeletonema costatum to Changing Ambient Phosphorus
Source: Front Microbiol. 2016 Sep 20;7:1476. doi: 10.3389/fmicb.2016.01476 (PMC5028394; doi:10.3389/fmicb.2016.01476)

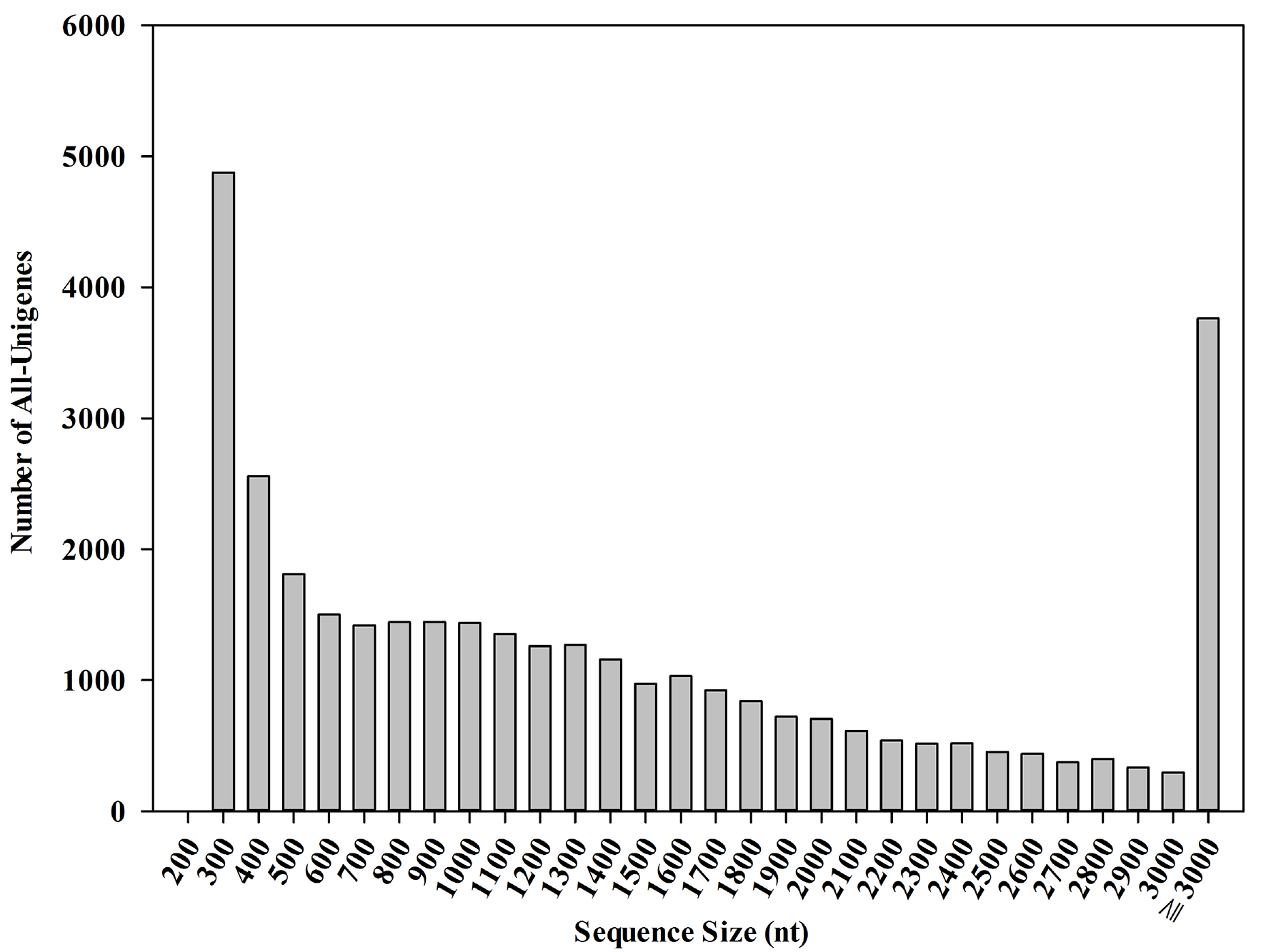

Supplement: Supplementary file 1 [file Presentation_1.ZIP › Supplementary Material/Fig.S1.tif]

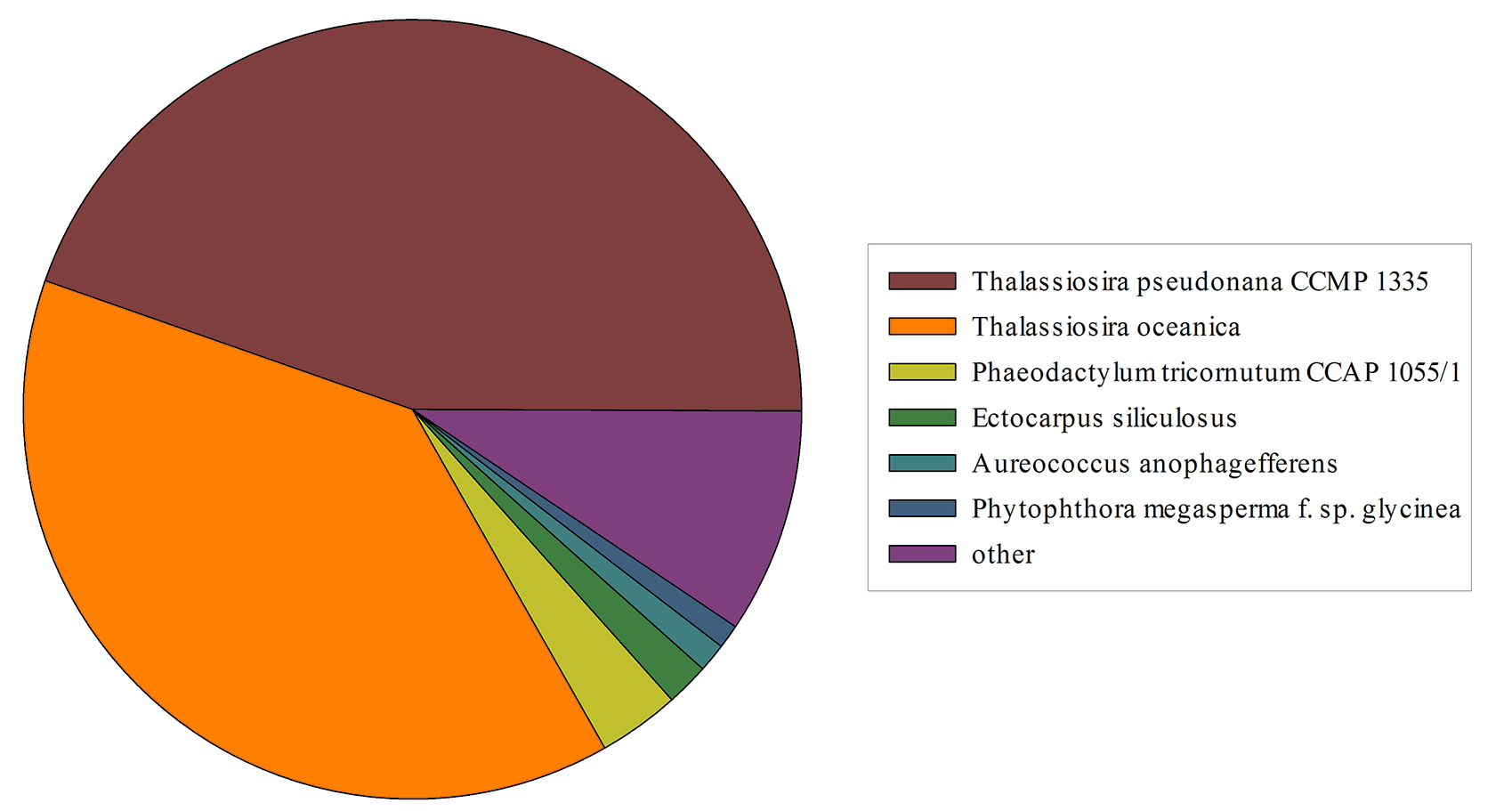

Supplement: Supplementary file 1 [file Presentation_1.ZIP › Supplementary Material/Fig.S2.TIF]

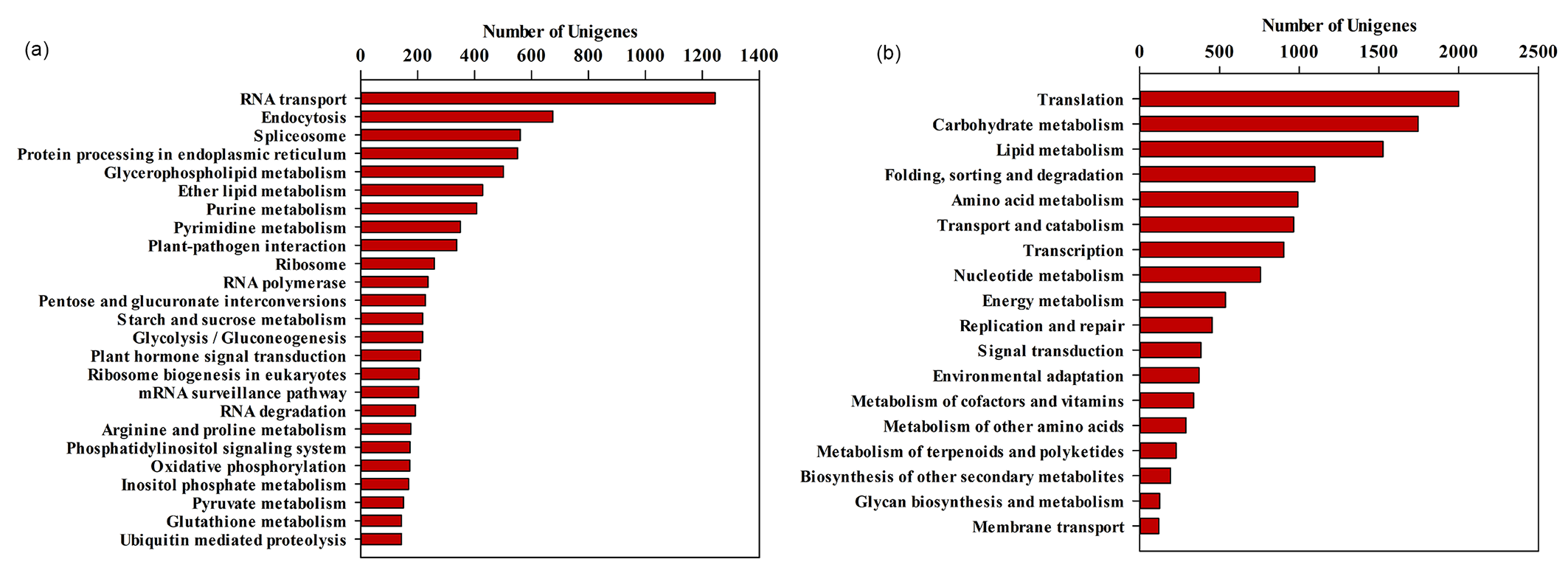

Supplement: Supplementary file 1 [file Presentation_1.ZIP › Supplementary Material/Fig.S3.tif]

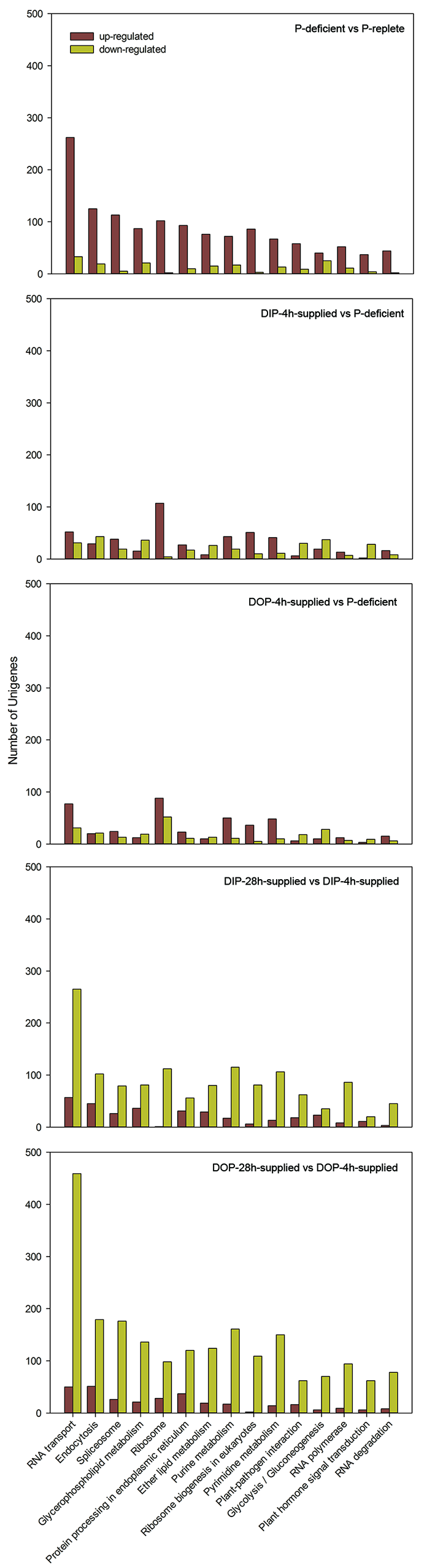

Supplement: Supplementary file 1 [file Presentation_1.ZIP › Supplementary Material/Fig.S4.tif]
