# Supplementary material for: Comparative Transcriptomic Analysis Reveals Novel Insights into the Adaptive Response of Skeletonema costatum to Changing Ambient Phosphorus
Source: Front Microbiol. 2016 Sep 20;7:1476. doi: 10.3389/fmicb.2016.01476 (PMC5028394; doi:10.3389/fmicb.2016.01476)
Supplement: Supplementary file 1 [file Presentation_1.ZIP › Supplementary Material/Supplementary Material.doc]

**Comparative Transcriptomic Analysis Reveals Novel**

**Insights into the Adaptive Response of *Skeletonema costatum* to Changing Ambient Phosphorus**

Shu-Feng Zhang, Chun-Juan Yuan, Ying Chen, Xiao-Huang Chen, Dong-Xu Li, Jiu-Ling Liu, Lin Lin, Da-Zhi Wang*

**Correspondence:** dzwang@xmu.edu.cn.

**Supplementary Figures and Tables**

**Supplementary Figures**

**Supplementary Fig. S1** Sequence size distribution of All-Unigenes assembled from Illumina reads. All the clean reads from the six samples were combined and gave rise to 34,942 unigenes.

**Supplementary Fig. S2** Source species distribution of All-Unigenes annotated against NCBI-NR database.

**Supplementary Fig. S3** KEGG pathway annotation and classification of all unigenes annotated. (a) Pathway enrichment analysis of all unigenes against KEGG database in Level 2. (b) Top 25 KEGG pathways with number of unigenes.

**Supplementary Fig. S4** Variations of top 15 enriched pathways against KEGG database in *S. costatum* responded to P-deficient and P-resupplied conditions.

**Supplementary Tables**

**Supplementary Table S1** Physiological/ecological relevance of each pair-wise comparison.

**Supplementary Table S2** Primers used for qRT-PCR in this study.

**Supplementary Table S3** Statistics of all differentially expressed gene pathway enrichment analysis against the KEGG database.

**Supplementary Table S4** DEGs related to P metabolism of *S. costatum* under different P conditions among P-replete, P-deficient and P-resupply, including raw fragments, FPKM and annotation.

**Supplementary Table S5** DEGs related to circadian rhythm in *S. costatum* under different P conditions among P-replete, P-deficient and P-resupply, including raw fragments, FPKM and annotation.

**Supplementary Table S6** DEGs related to other important metabolisms in *S. costatum* under different P conditions among P-replete, P-deficient and P-resupply, including raw fragments, FPKM and annotation.
